# Characterization and Identification of Lysine Succinylation Sites based on Deep Learning Method

**DOI:** 10.1038/s41598-019-52552-4

**Published:** 2019-11-07

**Authors:** Kai-Yao Huang, Justin Bo-Kai Hsu, Tzong-Yi Lee

**Affiliations:** 1 Department of Medical Research, Hsinchu Mackay Memorial Hospital, Hsinchu city, 300 Taiwan; 2 Department of Medical Research, Taipei Medical University Hospital, Taipei city, 110 Taiwan; 3 Warshel Institute for Computational Biology, The Chinese University of Hong Kong, Shenzhen, 518172 China; 4 School of Life and Health Sciences, The Chinese University of Hong Kong, Shenzhen, 518172 China

**Keywords:** Proteome informatics, Machine learning, Protein function predictions

## Abstract

Succinylation is a type of protein post-translational modification (PTM), which can play important roles in a variety of cellular processes. Due to an increasing number of site-specific succinylated peptides obtained from high-throughput mass spectrometry (MS), various tools have been developed for computationally identifying succinylated sites on proteins. However, most of these tools predict succinylation sites based on traditional machine learning methods. Hence, this work aimed to carry out the succinylation site prediction based on a deep learning model. The abundance of MS-verified succinylated peptides enabled the investigation of substrate site specificity of succinylation sites through sequence-based attributes, such as position-specific amino acid composition, the composition of *k*-spaced amino acid pairs (CKSAAP), and position-specific scoring matrix (PSSM). Additionally, the maximal dependence decomposition (MDD) was adopted to detect the substrate signatures of lysine succinylation sites by dividing all succinylated sequences into several groups with conserved substrate motifs. According to the results of ten-fold cross-validation, the deep learning model trained using PSSM and informative CKSAAP attributes can reach the best predictive performance and also perform better than traditional machine-learning methods. Moreover, an independent testing dataset that truly did not exist in the training dataset was used to compare the proposed method with six existing prediction tools. The testing dataset comprised of 218 positive and 2621 negative instances, and the proposed model could yield a promising performance with 84.40% sensitivity, 86.99% specificity, 86.79% accuracy, and an MCC value of 0.489. Finally, the proposed method has been implemented as a web-based prediction tool (CNN-SuccSite), which is now freely accessible at http://csb.cse.yzu.edu.tw/CNN-SuccSite/.

## Introduction

Post-translational modifications (PTMs), which are biochemical reactions occurring on proteins, have been known to have crucial roles in cellular processes such as DNA repair, transcriptional regulation, signaling pathways, protein–protein interactions, apoptosis, cell death, and metabolic pathways^1^. Protein succinylation is a type of PTM involving the attachment of a succinyl group (-CO-CH_2_-CH_2_-CO-) to a specific lysine residue of a protein^2^. Protein lysine succinylation, mediated by succinyl-coenzyme A (succinyl-CoA), has been identified to play crucial roles in regulating a variety of cellular processes^3,4^. In recent years, high-throughput mass spectrometry (MS) has been widely adopted to identify large-scale datasets of site-specific succinylation peptides^5–8^. Proteome-wide profiling analyses have revealed the involvement of succinylation in multiple metabolic pathways^8^ and cellular physiology^9^, especially for thermophilic and mesophilic bacteria^7^. In addition, a quantitative succinylome analysis in breast cancer expanded our understanding of mechanisms of tumorigenesis and provided further characterization of the pathophysiological roles of succinylation in breast cancer progression, which can enable innovative therapies for breast cancer patients^10^. However, the functions of protein succinylation in diseases and cancer are still not well studied. The limited number of studies involving functional investigations of protein succinylation has motivated us to provide a functional enrichment analysis for all succinylated proteins.

Due to the quantitative succinylome data obtained from MS-based proteomics techniques, a variety of bioinformatics tools have been developed for predicting lysine succinylation sites based on protein sequences. A list of previously proposed approaches concerning computational annotation of succinylated sites is given in Supplementary Table S1. SucPred^11^ is a succinylation site prediction tool designed by Zhao *et al*. based on support vector machine (SVM) with the consideration of multiple feature-encoding algorithms. SuccFind^12^ was developed based on sequence-derived features and evolutionary-derived sequence information with an enhanced feature optimization strategy. The iSuc-PseAAC was proposed by incorporating the peptide position-specific propensity into the general form of pseudo amino acid composition^13^. Hasan *et al*. have proposed a web server, named SuccinSite, that incorporates three sequence encodings — k-spaced amino acid pairs and binary and amino acid index properties — for predicting succinylated lysine sites^14^. A computational tool termed iSuc-PseOpt has been developed to predict protein succinylation sites by incorporating the sequence-coupling effects into the general pseudo amino acid composition and using K-nearest neighbors cleaning (KNNC) treatment and inserting hypothetical training samples (IHTS) treatment to optimize the training dataset^15^. In 2017, Hasan *et al*. further proposed the SccinSite 2.0 for a systematic identification of species-specific protein succinylation sites by using joint element features information^16^. Recently, a new method called Success was developed by integrating evolutionary and structural characteristics to provide accurate predictions of protein succinylation sites^17^. Lopez *et al*. also published another succinylation site prediction tool namely SSEvol-Suc^18^ in 2018. In October 2018, Hasan *et al*. published the GPSuc for a global prediction of generic and species-specific succinylation sites by aggregating multiple sequence features^19^. In 2019, Hasan *et al*. further proposed a large-scale assessment of prediction tools for lysine succinylation sites^20^.

Although many succinylation site prediction tools have been proposed, the performance of those approaches can still be improved. Moreover, the recent advancements of high-throughput techniques in biotechnology have identified more and more experimentally verified data of succinylation sites. The lack of deep learning-based approaches for identifying succinylation sites needed to be addressed. Therefore, we aimed to develop a new method for identifying protein succinylation sites based on a deep neural network^21^. In this work, four sequenced-based attributes, such as position-specific amino acid composition^22,23^, amino acid pairs composition^24–26^, position-specific scoring matrix (PSSM)^27^, and *k*-spaced amino acid pairs^28,29^, were considered for identifying protein succinylation sites. According to cross-validation evaluation, the model with the best cross-validation performance was further measured with an independent testing dataset.

## Material and Methods

**Figure 1:**
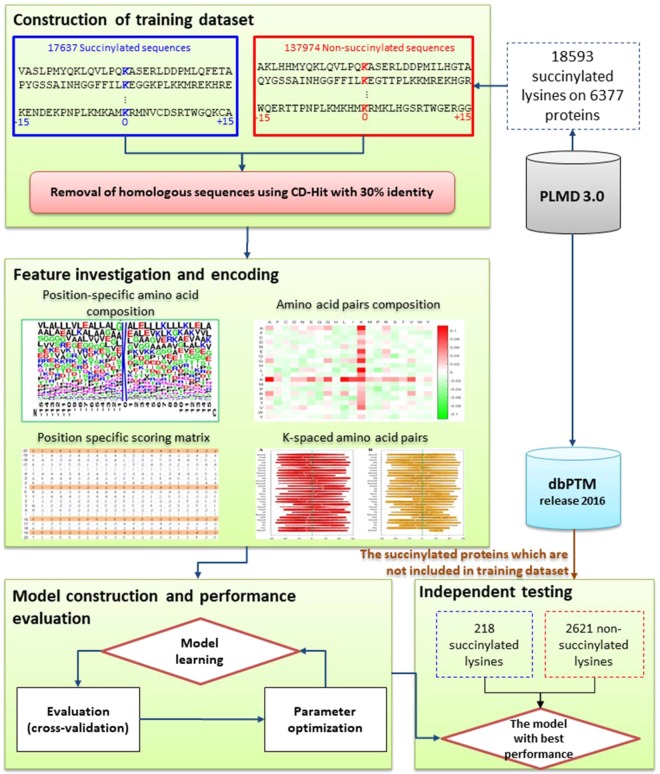
Flow chart of the proposed method. Four major steps were involved such as construction of training dataset, feature investigation and encoding, model construction and performance evaluation, and independent testing.

A flow chart of the proposed method is presented in Fig. 1, including (1) construction of the training dataset, (2) feature investigation and encoding, (3) model construction and performance evaluation, and (4) independent testing. First, the experimental data of known succinylated sites was mainly obtained from PLMD 3.0^30^. After constructing positive and negative training datasets, four different types of sequence-based encoding schemes were adopted to transform the sequences to multi-dimensional vectors. Then, ten-fold cross-validation was utilized to evaluate the performances of the predictive models trained based on deep convolutional neural networks. Finally, the model with the best predictive performance was further evaluated by an independent testing dataset, which was truly blind to the training dataset used for model construction. The detailed procedures are described in the following sections.

### Construction of positive and negative training datasets

In this study, the dataset of experimentally verified lysine succinylation sites was mainly extracted from PLMD^30^, which has accumulated 284,780 lysine modification sites from 53,501 proteins among 20 different types of PTMs. When considering only experimentally confirmed lysine succinylation sites, a total of 18,593 sites were obtained from 6,377 unique proteins. After the removal of homologous protein sequences, a (*2n* + 1)-mer window size was adopted to extract fragmented sequences centered on modified sites with *n* left-hand and *n* right-hand neighboring amino acids. Given a specific number of succinylated proteins, the negative dataset was generated from non-succinylated sites which are those fragmented sequences centered on lysine residues that lack succinylation annotation. By evaluating different values of window size (2*n* + 1, *n* is ranging from 5 to 20), the 31-mer window size (n = 15) performed best for predicting lysine succinylation sites (Supplementary Fig. S1), based on the basic feature—position-specific amino acid composition. After filtering out the fragmented sequences with sequence lengths less than 31 amino acids, a total of 17,637 and 137,974 fragmented sequences were retained for positive and negative training datasets, respectively. A training dataset with high sequence similarity will overestimate cross-validation performance^26,31–33^. Therefore, after the removal of the duplicated and homologous sequences by employing the CD-HIT program^34^ with 30% sequence identity, we obtained a total of 1,268 non-homologous succinylated proteins, which comprise 3,216 succinylated and 16,412 non-succinylated lysine residues for positive and negative training datasets, respectively. Table 1 provides the detailed statistics of positive and negative instances in accordance with various sequence identity thresholds of CD-HIT.

**Table 1:** Data statistics of positive and negative training datasets using CD-HIT with various values of sequence identity threshold.

Sequence identity threshold	Number of succinylated proteins	Number of succinylated lysine sites	Number of non-succinylated lysine sites
Full data	6,034	17,637	137,974
100%	5,539	15,691	117,813
90%	4,924	13,656	97,629
80%	4,422	12,031	86,192
70%	3,812	10,908	69,656
60%	3,105	8,855	51,456
50%	2,517	6,201	33,904
40%	1,869	4,509	23,577
30% (**Training data**)	1,268	3,216	16,412

### Feature investigation and encoding

This study aimed at the sequence-based characterization of protein succinylation site specificity. Not only the position-specific amino acid composition (PspAAC) but also the composition of *k*-spaced amino acid pairs (CKSAAPs) and position-specific scoring matrix (PSSM) were considered for use as the training attributes for constructing predictive models as well as measuring discriminating powers.

#### Position-specific Amino acid composition (PspAAC)

Amino acid composition (AAC) has been regarded as a typical attribute for examining substrate site motifs on a variety of PTMs^35–41^. AAC was defined to determine the probability of amino acids occurring in the flanking region of PTM sites. Since a training sequence *x* has a length of 31 amino acids, the probability *P*_*x*_(*k*) of a specific amino acid *k* was elaborated as^42^

1 $${P}_{x}\,(k)=\,\frac{{n}_{x}(k)}{{\sum }_{k=1}^{20}{n}_{x}(k)}\,k=1,2,\ldots ,20$$

where *n*_*x*_*(k)* represents the number of occurrences of a specific amino acid *k*. Refer to the method of positional weighted matrix (PWM) of amino acids around sulfation sites^43^, the position-specific amino acid composition (PspAAC) around the succinylated sites was determined using non-homologous training datasets. The PspAAC specified the relative frequency of twenty amino acids of each position that surrounded the succinylation sites, and was utilized in encoding the fragment sequences. A matrix of *m* × *w* elements was used to represent the PspAAC of a training dataset, where *m* stands for 20 types of amino acids and *w* is the window size ranging from −15 to +15. The matrix with 20 × 30 features was represented as:

2 $$PspAAC=[\begin{array}{ccc}{P}_{-15}(1) & \cdots  & {P}_{+15}(1)\\ \vdots  & \ddots  & \vdots \\ {P}_{-15}(m) & \cdots  & {P}_{+15}(m)\end{array}]$$

### Composition of k-spaced amino acid pairs (CKSAAP)

The composition of k-spaced amino acid pairs (CKSAAP) has been extensively applied in analyses of protein functions^28,33,41,44–49^. This study transformed all training sequences into numeric vectors based on the encoding method of CKSAAP. Given *k* values ranging from zero to five, the number of occurrence of each *k*-spaced AAP can be determined from target sequences. If *k* is set as one, $[{A}_{i}x{A}_{j}]$ was used to represent the pair of amino acids *A*_*i*_ and *A*_*j*_ (*i* and *j* = 1, …, 20, corresponding to 20 amino acids) which are separated by one residue of any amino acid *x*. If *k* is set as two, $[{A}_{i}xx{A}_{j}]$ represented the pair of amino acids *A*_*i*_ and *A*_*j*_ that are separated by two amino acids *xx*. The occurring count of a one-spaced AAP $[{A}_{i}x{A}_{j}]$ was represented by $N([{A}_{i}x{A}_{j}])$ and its conditional probability $P[{A}_{i}x{A}_{j}]$ was defined as:

3 $$P[{A}_{i}x{A}_{j}]=\frac{N([{A}_{i}x{A}_{j}])}{N([{A}_{i}x{A}_{\ast }])}$$

where $\,N([{A}_{i}x{A}_{\ast }])=\sum _{j=1,\,\ldots ,20}N([{A}_{i}x{A}_{j}])$. In order to identify the difference of occurring frequency of a KSAAP between positive and negative sequences, for instance, the diversity of a one-spaced AAP [*A*_*i*_*xA*_*j*_] can be obtained from:

4 $$C[{A}_{i}x{A}_{j}]=\,\log \,\frac{{P}^{+}[{A}_{i}x{A}_{j}]}{{P}^{-}[{A}_{i}x{A}_{j}]}$$

where ${P}^{+}[{A}_{i}x{A}_{j}]$ and ${P}^{-}[{A}_{i}x{A}_{j}]$ are the conditional probabilities of a one-spaced AAP $[{A}_{i}x{A}_{j}]$ in positive and negative training sequences, respectively. In this investigation, a higher positive value of $C[{A}_{i}x{A}_{j}]$ indicated that the one-spaced AAP $[{A}_{i}x{A}_{j}]$ is a more significant attribute in the positive dataset; otherwise, a smaller negative value of $C[{A}_{i}x{A}_{j}]$ revealed it is a more abundant attribute in negative dataset. Among a total of 2400 KSAAPs, we utilized a feature selection approach, minimum redundancy–maximum relevance (mRMR), to generate an index score for each KSPAAP^50^. A KSAAP with minimum redundancy and maximum relevance was regarded as the best attribute for classifying succinylated and non-succinylated sequences. The scoring function of mRMR was described as:

5 $$scor{e}_{j}=M({f}_{j},c)-\frac{1}{m}\mathop{\sum }\limits_{i=1}^{m}M({f}_{i},{f}_{j}),$$

where in ${f}_{j}\subset {S}_{n}$, ${f}_{i}\subset {S}_{m}$, ${S}_{m}=S-{S}_{n}$, in which *S*_*m*_, *S*_*n*_, and *S* were the attribute sets (*m* and *n* were the attribute sizes), and *c* is a classification variable with two possible classes. Additionally, the mutual information $M(x,y)$ was defined as:

6 $$M(x,y)=\iint p(x,y)\log \,\frac{p(x,y)}{p(x)p(y)}dxdy,$$

where $p(x,y)$, $p(x)$, and $p(y)$ were regarded as the probabilistic density functions between attributes *x* and *y*. In addition, the sequential forward selection (SFS) was employed to a final set of 400 most discriminating KSAAPs according to the ranking of mRMR index scores.

#### Position specific scoring matrix (PSSM)

**Figure 2:**
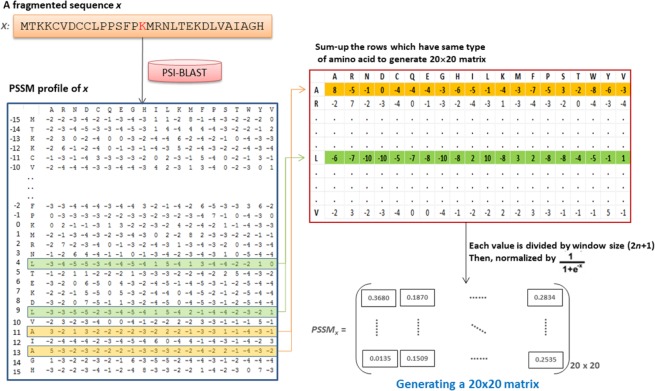
Flow chart of generating a 20 × 20 matrix based on the PSSM profile obtained from PSI-BLAST.

From a structural viewpoint, several amino acid residues can be mutated without changing a protein’s tertiary structure, and two proteins may have similar structures with different amino acid compositions^51^. PSSM profiles, which have been extensively utilized in protein secondary structure prediction, subcellular localization, and other bioinformatics analyses^51–54^, were adopted herein with significant improvement. As presented in Fig. 2, the PSSM profile of each training sequence was generated by performing PSI-BLAST^55^ against the database of non-homologous succinylated peptides. The PSSM profile was composed of a matrix with *w* × *m* elements, where *w* stands for the sequence length (ranging from −15 to +15) and *m* represents 20 types of amino acids, which is row-centered at modified site.

7 $$Profil{e}_{x}=[\begin{array}{ccc}{p}_{x,-15}(1) & \cdots  & {p}_{x,-15}(m)\\ \vdots  & \ddots  & \vdots \\ {p}_{x,+15}(1) & \cdots  & {p}_{x,+15}(m)\end{array}]$$

Then, the *w* × *m* matrix was transformed into a matrix with 20 × 20 features *S*_*x*_ (*i*, *j*), where *i* and *j* range from 1 to 20, by summing up the rows that were involved in the same type of amino acid *i*.

8 $$PSS{M}_{x}=[\begin{array}{ccc}{S}_{x}(1,1) & \cdots  & {S}_{x}(1,20)\\ \vdots  & \ddots  & \vdots \\ {S}_{x}(20,1) & \cdots  & {S}_{x}(20,20)\end{array}]$$

Finally, each element in the 20 × 20 matrix was divided by the window length *w* and then normalized using a sigmoid function:

9 $${N}_{x}(i,j)=\frac{1}{1+{e}^{\frac{-{S}_{x}(i,j)}{w}}}i,j=1,\ldots ,20$$

### Characterization of substrate site signatures

To investigate into the substrate-site specificity of succinylated sites, the maximal dependence decomposition (MDD)^40^ was employed to divide positive training sequences into several groups with potentially conserved motifs. The MDD has been reported to having the ability to enhance the predictive effectiveness of computationally identifying substrate sites on different PTM types^31,35,56^. For reaching this purpose, a chi-squared test ${{\rm{\chi }}}^{2}({P}_{{\rm{i}}},{P}_{{\rm{j}}})$ is adopted to examine the intrinsic interdependence between two positions, ${P}_{{\rm{i}}}$ and ${P}_{{\rm{j}}}$, which are in the neighboring upstream and downstream regions of succinylation sites. Amino acids, 20 in total, are categorized into five groups, based on their physicochemical properties: polar, acidic, basic, hydrophobic, and aromatic. Given two positions ${P}_{{\rm{i}}}$ and ${P}_{{\rm{j}}}$, the occurring frequency of the presence of each amino acid group is determined for the elements of a contingency table. The chi-squared test is defined as:

10 $${{\boldsymbol{\chi }}}^{2}({{\boldsymbol{P}}}_{{\boldsymbol{i}}},{{\boldsymbol{P}}}_{{\boldsymbol{j}}})=\mathop{\sum }\limits_{{\boldsymbol{m}}=1}^{5}\mathop{\sum }\limits_{{\boldsymbol{n}}=1}^{5}\frac{{({{\boldsymbol{K}}}_{{\boldsymbol{mn}}}-{{\boldsymbol{Q}}}_{{\boldsymbol{mn}}})}^{2}}{{{\boldsymbol{Q}}}_{{\boldsymbol{mn}}}}$$

where ${K}_{mn}$ is the number of positive training sequences containing amino acids in group *m* at position ${P}_{i}$ and containing amino acids in group $n$ at position ${P}_{j}$, for each pair $({P}_{i},{P}_{j})$ and $i\ne j$. The expectation value ${Q}_{mn}$ is obtained from

11 $${Q}_{mn}=\frac{{K}_{nR}-{K}_{Cn}}{K}$$

where ${K}_{mR}={K}_{m1}+\ldots +{K}_{m5}$, ${K}_{Cn}={K}_{1n}+\ldots +{K}_{5n}$ and *K* stands for the total number of positive training sequences. The ${{\rm{\chi }}}^{2}({P}_{{\rm{i}}},{P}_{{\rm{j}}})$ is a significant dependence if its value is larger than 34.3, based on the p-value of 0.01 with degree of freedom at 16^57^. When performing MDD on the dataset of all positive training sequences, the parameter of maximum cluster size should be specified with an appropriate cutoff value. The MDD clustering process will be terminated when all the group sizes are less than the specified value of maximum cluster size.

### Construction of deep neural networks

**Figure 3:**
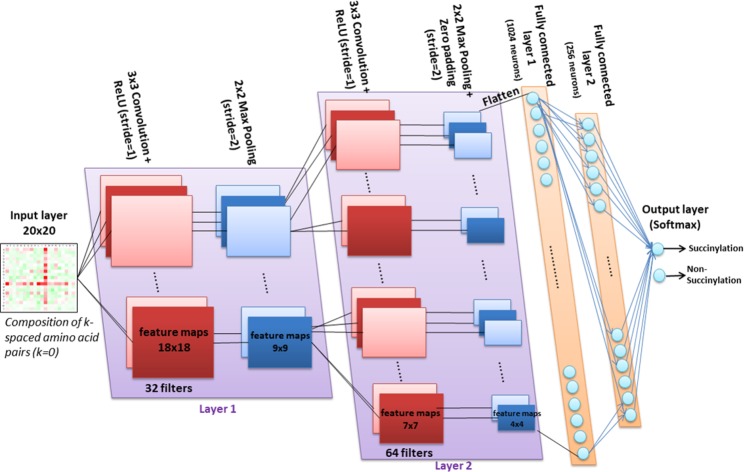
Schematic diagram of incorporating deep convolutional neural network with CKSAAP attribute (*K* = 0) to learn a predictive model with two-node output layer. A total of eight layers were implemented in this work, such as one input layer, two convolution layers, two max pooling layers, two fully connected layers, and one output layer. For each dense layer, the ReLU activation function was applied to avoid gradient diffusion. In addition, the dropout step was conducted in the hidden layers with an attempt to reduce overfitting. Finally, the output layer is composed of two nodes corresponding to the classifying results based on a softmax function.

This study involved a binary classification of lysine residues into succinylated and non-succinylated sites. Due to the emergence of applying deep learning methods in bioinformatics^58^, we utilized a deep convolutional neural network (CNN), which is an extension of an artificial neural network (ANN) with multiple hidden layers between input and output layers (Supplementary Fig. S2). With the increasing number and complexity of high-throughput biological datasets, CNNs can decipher more complicated patterns and relationships within the investigated attributes than a traditional ANN, which only includes one hidden layer. A significant increase in the data count of MS/MS-identified protein succinylation would enable the number of neurons required in each layer to increase exponentially along with the potential patterns. Hence, this work exploited a CNN to learn the predictive models using various types of sequence-based attributes. In recent years, CNNs have been extended to incorporate convolution and pooling strategies in hidden layers to reduce the quantity of weights and complexity of calculations, respectively, when generating network structure. When implementing a CNN model, it is necessary to determine the number of convolution and pooling layers and choose a classification function for the output layer. As presented in Fig. 3, the first layer of CNN is the input layer. The AAPC attribute, represented as a matrix with 20 × 20 elements, was used as an example for constructing the CNN model.

When developing a CNN model, the convolution layer is the core layer, which functions as a pattern scanner and contains two major parameters: filters (or kernels) and stride. Each filter, which can be regarded as a small pattern with specified matrix size (e.g. 3 × 3 used in this work), is convolved across the width (20) and height (20) of the input data, based on the dot product between the elements of the filter and the input data in order to create new feature maps. We specified the value of stride as 1, then moved the filter one pixel at a time, and the input data with a 20 × 20 matrix size can be transformed into a new feature map with a matrix size of (20–3 + 1) × (20–3 + 1). The number of filters controls the depth (the number of neurons) in the convolution layer that may detect a specific type of pattern connecting to the input data. In addition to filters and stride, zero padding is a convenient approach to pad the input with zeros on the border of the matrix. Zero padding can be used to control the matrix size of input data.

The pooling layers, which comprise another critical part of a CNN model, usually immediately follow the convolution layers. Max pooling is a sort of non-linear down-sampling strategy used frequently for CNN construction. Typically, the max pooling layer can split the input matrix into a set of non-overlapping rectangles and can form a smaller matrix containing maximal outputs of each sub-region. Two major parameters used in max pooling are kernel size and stride, which are usually set as 2 × 2 and 2, respectively, for moving the 2 × 2 kernel along width or height 2 pixels at a time, discarding 75% of the activations. For instance, a feature map with matrix size of 18 × 18 in the convolution layer can be transformed into a smaller feature map with matrix size of 9 × 9 in the following max pooling layer. The function of max pooling is to reduce the amount of computing time in a CNN model and examine if the patterns extracted from the corresponding convolution layer exist in the input data or not^59^.

After two convolution and max pooling layers, the highly complicated CNN modeling was accomplished by fully connected layers. Before getting into the fully connected layer, the flattening step (flatten layer) is a necessity that can be used to convert the matrix of input data into a vector. The flattening process is typically used prior to the fully connected layer. In a general CNN model, neurons in a fully connected layer have full links to all activations in the previous layer, as shown in Fig. 3. Thus, all the activations in the previous layer can be summarized by matrix multiplication along with a set of weight values on the links. Due to the occupation of most neurons in fully connected layers, an over-fitting problem might easily occur during CNN model construction. Herein, the dropout layer has been adopted to randomly mask a specified portion of its neurons in order to prevent CNN model construction from an over-fitting problem^60^. The dropout layer is carried out by dropping out the neurons with a specified probability *P* and retaining the neurons with probability 1 – *P*. The value of probability *P* ranges from 0 to 1 with an attempt to determine the best *P* value for optimizing predictive performance. After that, we obtained a reduced network, in which the incoming and outgoing links to the dropped-out neurons are also eliminated.

As for the binary classification between succinylated and non-succinylated sites, the output layer comprised two neurons corresponding to the classification results based on a softmax function. The two nodes in the output layer were fully connected to the neurons of the previous layer. The softmax function could be regarded as a loss function by specifying how to penalize the difference between the predicted and true classes. The softmax function (or normalized exponential function) is a kind of logistic function that can be used to represent a probability distribution over *K* different categories. In this work, the value of *K* was set as two for the succinylated and non-succinylated datasets. Given a sample vector *x* and a weight vector *w*, the predicted probability for *j*-th class by the softmax function is defined as

12 $${\rm{P}}({\rm{class}}={\rm{j}}/x)=\frac{{e}^{{x}^{T}{w}_{j}}}{{\sum }_{i=1}^{K}{e}^{{x}^{T}{w}_{i}}},j=1\,{\rm{or}}\,2$$

This can be regarded as the probability of x for the *j*-th class against the composition of *K* linear functions: ${x}^{T}{w}_{i},\,i=1,\ldots ,\,K$. Additionally, the ReLU is frequently used as the activation function when generating a CNN model with an enhanced nonlinear property but without a significant penalty for generalization accuracy^61^. In this work, the ReLU function was also employed to avoid gradient diffusion during the process of CNN construction. The ReLU function is defined as: ${\rm{ReLU}}(x)=\,{\rm{\max }}(0,x)$. Another two activation functions are the sigmoid function ${\rm{\sigma }}(x)=\frac{1}{1+{e}^{-x}}$ and the hyperbolic tangent function $\tanh (x)=\frac{{e}^{x}-{e}^{-x}}{{e}^{x}+{e}^{-x}}$.

### Performance evaluation of predictive models

In the generation of CNN models, the *k*-fold cross-validation was employed to evaluate their predictive performances. When implementing *k*-fold cross-validation, all the training data, including positive and negative sequences, were randomly clustered into k equal-sized subgroups. After having k subgroups, k-1 of them shall be regarded as the training sample and the remaining one subgroup was considered as the validation sample. In a round of k-fold cross-validation, each of the k subgroups should be considered as the validation sample once in turn. Sensitivity (*Sn*), specificity (*Sp*), accuracy (*Acc*), and Matthews correlation coefficient (MCC) have been used as the metrics to determine the performance of the generated models. The four metrics are defined as:

13 $$Sn=\frac{TP}{TP+FN}$$

14 $$Sp=\frac{TN}{TN+FP}$$

15 $$Acc=\frac{TP+TN}{TP+FP+TN+FN}$$

16 $$MCC=\frac{(TP\times TN)-(FN\times FP)}{\sqrt{(TP+FN)\times (TN+FP)\times (TP+FP)\times (TN+FN)}}$$

where TP, FN, TN, and FP denote the instances of true positive, false negative, true negative, and false positives, respectively. Due to the unbalanced positive and negative training datasets in this work, we have decided to choose MCC value as a major benchmark for achieving a relatively balanced sensitivity and specificity. After evaluating the of *k*-fold cross-validation, the CNN model reaching the best predictive performance was further evaluated by an independent testing dataset that was not included at all in the training dataset.

### Independent testing

Due to the potential over-fitting issue originating from the training dataset, the predictive power of the generated models might be overestimated. Thus, the use of an independent testing dataset was necessary to further evaluate of the real case. In this study, the independent testing dataset was mainly collected from dbPTM^44,45,62^. Before the extraction of positive and negative testing sequences, the experimentally verified succinylated proteins in testing dataset were compared with training dataset in order to eliminate the homologous protein sequences between the two datasets. When extracting sequence fragments using the same window length as used in constructing the training dataset, the fragmented sequences might be overlapped between the two datasets. Hence, CD-HIT software was used again to delete fragmented sequences with 30% similarity. After that, the final dataset for independent testing contained 218 succinylated and 2621 non-succinylated entries. Moreover, the testing dataset was utilized to make a comparison between the proposed deep-learning models and other machine learning schemes in terms of predictive performance. Another cause of over-fitting might be due to the training process of the CNN. To avoid the over-fitting problem, we only used two convolution layers with lower filters to reduce the complexity of our model by minimizing the possible training parameters^59^.

## Results and Discussion

### Substrate site signatures of lysine succinylation

**Figure 4:**
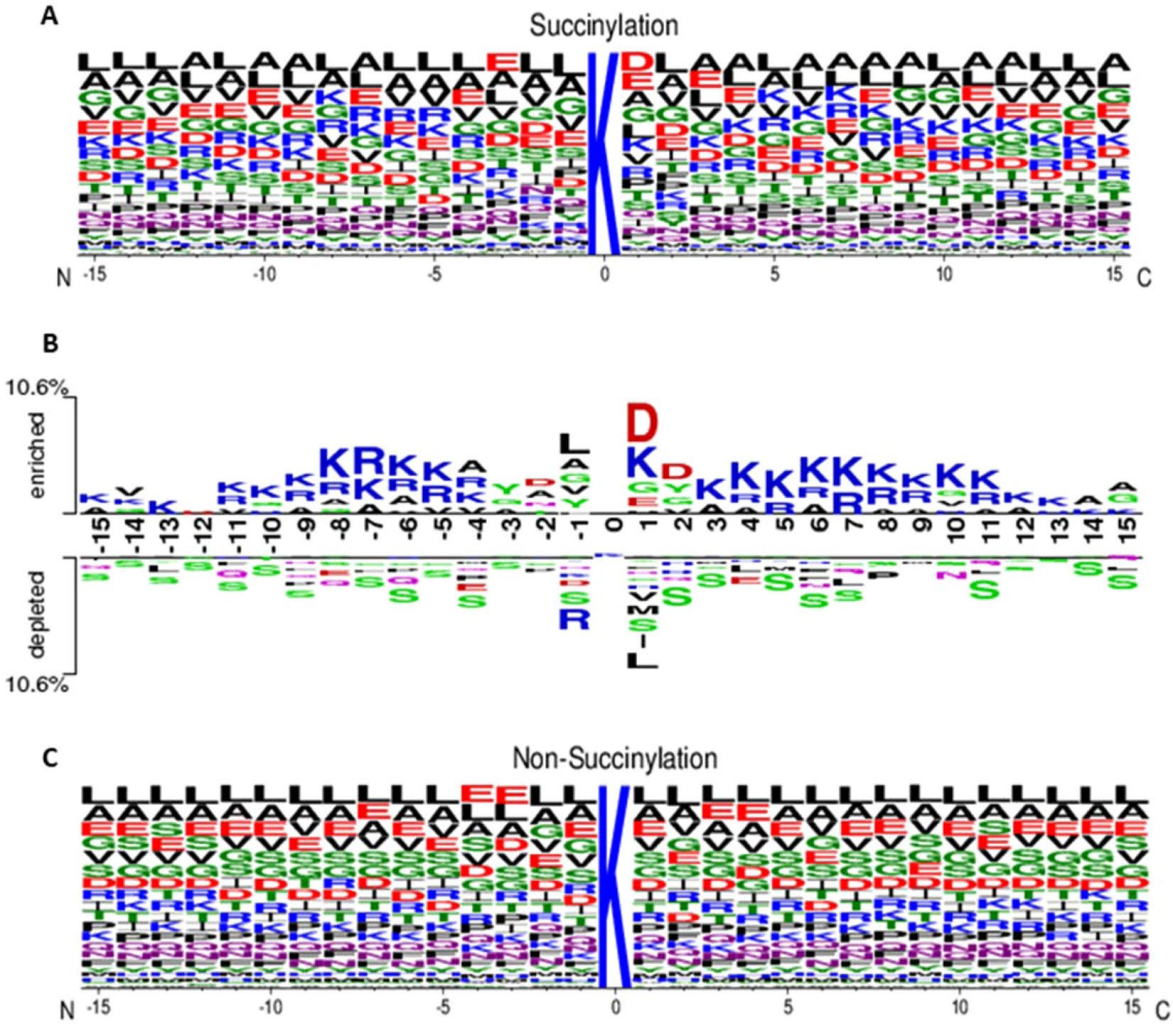
Position-specific amino acid composition of succinylated sites. (**A**) Position-specific amino acid composition of succinylated sequences based on the frequency plot of WebLogo. (**B**) Comparison of position-specific amino acid composition between succinylated and non-succinylated sequences based on TwoSampleLogo analysis. (**C**) Position-specific amino acid composition of non-succinylated sequences based on the frequency plot of WebLogo.

The amino acid composition (AAC) was a feasible scheme to explore the potential motif of conserved residues around the succinylation sites based on the fragments with 31-mer sequence length. Since comparing the AAC between positive and negative datasets, the residues having significant differences could be regarded as useful attributes for succinylated sites prediction. Supplementary Fig. S3 showed that, for succinylated sites, the positively charged lysine (K) residue appeared to have the highest frequency around the substrate sites. In addition to AAC, the position-specific AAC neighboring the succinylation sites can be displayed by frequency plots of WebLogo^63^. As illustrated in Fig. 4A, there is no any amino acid having significantly high frequency near the succinylation sites, but the slightly prominent amino acid residues included Leucine (L), Lysine (K), Alanine (A), and Valine (V). Without conserved motifs observed in frequency plot, the TwoSampleLogo^64^ program was further applied to compare the differences of position-specific AAC between succinylated and non-succinylated sequences. As displayed in Fig. 4B, when comparing with the sequence logo of non-succinylated sites (Fig. 4C), the most conserved motifs appeared to be associated with charged residues, in particular the positively charged K and arginine (R) residues on positions −11~−4 and +3~+12. Additionally, the negatively charged amino acids, such as aspartic acid (E), located at positions −2, +1 and +2.

**Figure 5:**
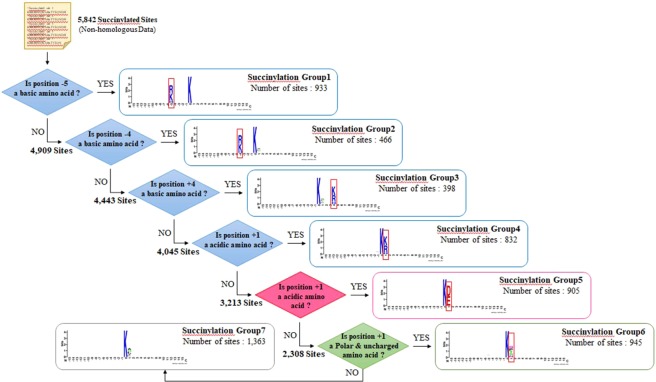
A hierarchical MDD-clustering process on the detection of motif signatures from 5842 succinylated sequences.

A hierarchical clustering analysis was performed on the detection of motif signatures by categorizing all positive training sequences into seven subgroups that possess statistically significant dependencies of amino acid composition around the substrate sites. The MDD-clustered subgroups with motif signatures for the 5842 non-homologous succinylated sites are presented in Fig. 5 based on a tree-like structure. The motif in Group1 (933 sequences) is the significant occurrence of basic amino acids (K, R, and H) at position −5, with the highest dependence value among all subgroups. In the meantime, the remaining 4909 sequences are further analyzed based on the maximal dependency in the occurrence of amino acids neighboring the substrate sites. The Group2 (466 sequences) possesses a similar motif of basic amino acids at position −4. Additionally, the Group3 (398 sequences) and Group4 (832 sequences) also have the motif of basic amino acids at position +4 and +1, respectively. This investigation demonstrates that the detected motif signatures are consistent with the observation in two-sample logo, which having positively charged residues conserved in the upstream and downstream regions of succinylated sites. On the other hand, the Group5 (905 sequences) has the conserved motif of acidic residues at position +1. The Group6 also reveals that the position +1 is potent that contains the motif signature of polar and uncharged amino acids. The remaining data in the Group7 contain a slightly significant character in position +1.

### Performance evaluation of CNN models trained with single attributes

In an attempt to examine the optimal window size for yielding the best performance, various window size values were adopted to extract the training sequences for model construction. After comprehensive analyses of performance comparisons, the window size of 31 (−15 to +15; with the succinylated residue in the center) achieved the best prediction performance, which is consistent with the difference of position-specific AACs between positive and negative training sequences. Based on the investigated features, their corresponding CNN models were built to determine the effectiveness of those features in identifying succinylation sites. As shown in Table 2, the CNN model trained with PspAAC reached an accuracy of 73.36% and an MCC value of 0.371. The AAPC model performed slightly better than the PspAAC model, which yielded an accuracy of 76.48% and an MCC value of 0.428. In the investigation of k-spaced amino acid pairs, the CNN model trained with the composition of one-spaced amino acid pairs (K = 1) provided the best performance at 77.95% sensitivity, 76.63% specificity, 76.85% accuracy, and MCC value of 0.432. After extracting the top 400 k-spaced amino acid pairs (K = 1−5) based on mRMR, the performance of the CNN model trained with the selected CKSAAP (top400) showed remarkable improvement, reaching a sensitivity of 85.35%, specificity of 83.49%, accuracy of 83.79%, and MCC value of 0.569. Among these CNNs, the model trained with the PSSM feature performed best for discriminating between succinylated and non-succinylated lysine residues. The PSSM model yielded a sensitivity, specificity, accuracy, and MCC value of 85.51%, 84.16%, 84.38%, and 0.579, respectively. Additionally, the ROC curve was generated to compare the predictive performance and stability of different CNN models (Supplementary Fig. S4). Regarding to the comparison among single features, the CNN model trained from the PSSM feature gave the best predictive power, which is consistent with the results reported in PSSM-Suc^65^. The area under ROC curve (AUC) of the CNN model trained with PSSM is 0.858. However, our investigation found that the CNN model trained with the composition of selected *k*-spaced amino acid pairs is comparable to that trained with the PSSM attribute.

**Table 2:** Evaluation of ten-fold cross-validation on deep learning models trained with various types of sequence-based attributes.

Attribute	Number of true positives	Number of false positives	Number of true negatives	Number of false negatives	Sensitivity	Specificity	Accuracy	MCC
PspAAC	2400	4411	12001	816	74.63%	73.12%	73.36%	0.371
CKSAAP (K = 0)	2512	3912	12500	704	78.11%	76.16%	76.48%	0.428
CKSAAP (K = 1)	2507	3835	12577	709	77.95%	76.63%	76.85%	0.432
CKSAAP (K = 2)	2501	3832	12580	715	77.77%	76.65%	76.83%	0.431
CKSAAP (K = 3)	2512	3912	12500	704	78.11%	76.16%	76.48%	0.428
CKSAAP (K = 4)	2494	3890	12522	722	77.55%	76.30%	76.50%	0.425
CKSAAP (K = 5)	2489	4079	12333	727	77.39%	75.15%	75.51%	0.412
CKSAAP (top400)	2745	2710	13702	471	85.35%	83.49%	83.79%	0.569
PSSM	2750	2600	13812	466	85.51%	84.16%	84.38%	0.579
PSSM + PspAAC	2759	2560	13852	457	85.79%	84.40%	84.63%	0.584
PSSM + CKSAAP (top 400)	2796	2391	14021	420	86.94%	85.43%	85.68%	0.608
PSSM + PspAAC + CKSAAP (top 400)	2789	2454	13958	427	86.72%	85.05%	85.32%	0.600

### Performance evaluation of CNN models trained with hybrid attributes

In addition to the comparison of predictive powers among single attributes, we also consider a hybrid of multiple attributes to generate the predictive model. Based on the results of performance testing of single attributes, the PSSM, which can yield the best performance, was selected as the principal attribute for the combination with other single attributes. Consequently, a total of three hybrids, such as PSSM + PspAAC, PSSM + CKSAAP(top400), and PSSM + PspAAC + CKSAAP(top400), were further evaluated for uncovering their predictive capabilities in the succinylation site identification. As presented in the Table 2, the CNN model trained using the hybrid of PSSM and PspAAC attributes can reach a comparable performance with that trained using single PSSM attribute. In this investigation, the CNN model trained using the hybrid of PSSM and CKSAAP (top400) could perform best with the sensitivity of 86.94%, the specificity of 85.43%, the accuracy of 85.68%, and the MCC value of 0.608. However, the CNN model trained with the combination of all features performs slightly worse than that trained with the hybrid of PSSM and CKSAAP (top400). Additionally, Supplementary Fig. S4 revealed that the CNN model trained using the hybrid of PSSM and CKSAAP (top400) can outperform other CNN models in terms of ROC curves comparison. The AUC value of the CNN model trained with PSSM and CKSAAP (top400) is 0.886.

### Performance comparison between CNN and other machine learning methods

To demonstrate the effectiveness of the deep learning method in PTM prediction, the predictive performance of this CNN model was compared with that of three popular machine learning methods: decision tree (DT), support vector machine (SVM), and random forest (RF). As summarized in Supplementary Table S1, the SVM and RF algorithms have been widely utilized to identify protein succinylation sites. In this work, the Classification and Regression Trees (CART) was employed to generate binary DTs for classifying between positive and negative instances. Based on the scikit-learn package^66^, the function ‘*DecisionTreeClassifier’* was used to construct a classification tree by a top-down recursion. During the construction process, the ‘best’ feature set was selected to classify the training tuples that make a split in the tree. In addition, the CART program specified the ‘Gini index’ as the feature set selection approach. For the construction of RFs, the CART was again adopted to generate multiple trees with the ‘bootstrap aggregation’ (bagging) of data sampling. In scikit-learn package, the function ‘*RandomForestClassifier’* was applied to measure the importance of training features and to generate the RF models. More specifically, Gini importance is the average decreased impurity of each feature across all trees; this impurity was the least-randomness of the given data. Moreover, the function ‘*svm.SVC’* in the scikit-learn package was used to train the binary SVM classifiers. The ‘radial basis function’ (RBF) was selected as the kernel function of SVM to transform the training data into a higher-dimensional vector space, with an attempt to search for a linearly optimal separating hyperplane.

According to the predictive performance of previous studies that have incorporated SVM or RF into their model construction, the SVM or RF models trained with combinatorial attributes could perform with reliable prediction accuracies. Based on the evaluation of ten-fold cross-validation, among the sequence-based attributes, this investigation has revealed that the DT model trained with PspAAC performed better than other attribute types (Supplementary Table S2). Instead of the PSSM attribute, both SVM and RF methods could reach a better performance by using the composition of the top 400 *k*-spaced amino acid pairs. Herein, the RF model performs slightly better than the SVM model in terms of MCC value. In addition to the comparison of different models trained using single attribute type, a hybrid of multiple attribute types was further considered into the generation of predictive models. Table 3 shows the comparison of ten-fold cross-validation between deep learning method and other three learning methods, on the basis of combining various attributes. Based on these sequence-based features, this investigation revealed that the CNN model trained using PSSM and CKSAAP(top400), which can yield the sensitivity, specificity, accuracy, and MCC values at 86.94%, 85.43%, 85.68%, and 0.608, respectively, can outperform other three learning methods. However, it is noteworthy that the RF model trained using the hybrid of PspAAC, PSSM, and CKSAAP(top400) attributes can yield a comparable performance (83.08% accuracy) to the CNN model. In conclusion, the proposed CNN model can outperform other three popular machine-learning methods, with reference to the comparison of predictive performances based on the evaluation of ten-fold cross-validation.

**Table 3:** Comparison of ten-fold cross-validation between deep learning method and other machine learning methods.

Method	Attribute	Number of true positives	Number of false positives	Number of true negatives	Number of false negatives	Sensitivity	Specificity	Accuracy	MCC
Decision tree	PspAAC + CKSAAP(top 400)	2282	4435	11977	934	70.96%	72.98%	72.64%	0.343
Support vector machine	PSSM + CKSAAP(top 400)	2622	3211	13201	594	81.53%	80.44%	80.61%	0.502
Random forest	PspAAC + PSSM + CKSAAP(top 400)	2605	2710	13702	611	81.00%	83.49%	83.08%	0.537
Deep learning	PSSM + CKSAAP(top 400)	2796	2391	14021	420	86.94%	85.43%	85.68%	0.608

### Performance evaluation using an independent testing dataset

When discriminating between succinylated and non-succinylated sequences, it is possible to generate a predictive model whose prediction accuracy is over-estimated due to an over-fitting problem. To avoid presenting an over-estimating performance, this work compiled a dataset for independent testing. These independent testing instances, which are not present in the training dataset, were used to measure the real ability of the proposed model. The independent testing dataset comprised a total of 218 positive and 2621 negative instances. The CNN model trained using the PSSM and CKSAAP(top400) attributes can yield a promising performance with a sensitivity of 84.40%, specificity of 86.99%, accuracy of 86.79%, and MCC value of 0.489. Additionally, to judge the practicality of the proposed model, the comparison between our model and six existing prediction tools was performed using the testing dataset. As displayed in Table 4, our proposed model achieved the highest MCC value, reaching 0.489. In this comparison, the SuccinSite 2.0 can provide the best predictive accuracy (88.83%), while its specificity (91.22%) was much higher than its sensitivity (60.09%). However, the overall performance of SuccinSite 2.0 did not outperform our method in terms of MCC value. Interestingly, as presented in Supplementary Fig. S5, most of the existing prediction tools can provide much better specificity values than sensitivity values. This might be because their models were generated by using the unbalanced positive and negative datasets. In an overall evaluation, the testing results have indicated that the proposed method can provide a more reliable and stable prediction capability than other existing prediction tools, in terms of balanced sensitivity and specificity.

**Table 4:** Performance comparison between our method and six existing available prediction tools based on the independent testing dataset.

Method	Number of true positives	Number of false positives	Number of true negatives	Number of false negatives	Sensitivity	Specificity	Accuracy	MCC
iSuc-PseAAC	31	310	2311	187	14.22%	88.17%	82.49%	0.019
SuccFind	101	921	1700	117	46.33%	64.86%	63.44%	0.062
pSuc-Lys	112	421	2200	106	51.38%	83.94%	81.44%	0.241
SuccinSite	98	221	2400	120	44.95%	91.57%	87.99%	0.308
SuccinSite 2.0	131	230	2391	87	60.09%	91.22%	88.83%	0.410
GPSuc	159	380	2241	59	72.94%	85.50%	84.54%	0.397
Our method	184	341	2280	34	84.40%	86.99%	86.79%	0.489

### Implementation of web-based prediction tool

**Figure 6:**
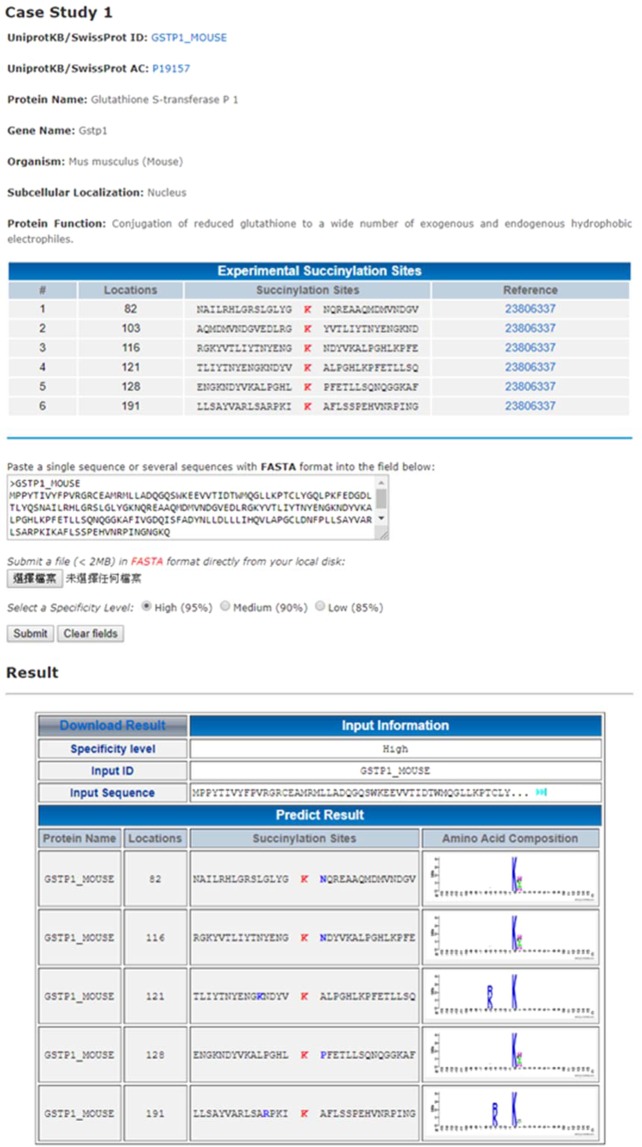
Case study of succinylation site prediction on Glutathione S-transferase P 1 (Gstp1).

To facilitate the functional analyses of protein succinylation, the proposed method has been utilized to implement a web-based tool, named CNN-SuccSite, for classifying between succinylated and non-succinylated sites. After submitting protein sequences in the FASTA format, the CNN-SuccSite will return the prediction results, including succinylated sites, their flanking amino acids, and the corresponding substrate motif signatures. A case study of succinylation site prediction on mouse Glutathione S-transferase P 1 (Gstp1) was utilized to demonstrate the effectiveness of CNN-SuccSite. The Gstp1 contains six verified succinylation sites at Lys-82, Lys-103, Lys-116, Lys-121, Lys-128, and Lys-191^67^. As presented in Fig. 6, the CNN-SuccSite can achieve an accurate prediction at five validated succinylaion sites, according to the corresponding motif signatures.

## Conclusion

Due to the abundance of experimentally verified succinylation data obtained from public resources, we were motivated to develop a new method to predict protein succinylation sites based on a deep learning strategy. Systematic investigation of various attributes in the neighborhood of substrate sites were performed on large-scale succinyl-proteome data. In accordance with the results of 10-fold cross-validation, the CNN model trained with the hybrid of PSSM and CKSAAP(top400) attributes can outperform that trained with other attributes. Besides, this investigation also demonstrated that the CNN model could provide a better performance than three popular shallow machine learning methods, including DT, SVM, and RF. Moreover, the independent testing was performed and the results demonstrated that the selected CNN model could outperform other existing prediction tools. Based on the usage of the independent testing dataset, the CNN model trained with the hybrid of PSSM and CKSAAP(top400) attributes could yield a promising performance. We truly believe that our proposed approach will help facilitate the determination of succinylated lysine residues of proteins. In the future, the physicochemical properties, such as solvent accessibility^68^, hydrophobicity^69^, and side-chain orientation^70^, can be considered for obtaining a better predictive performance. Additionally, the tertiary structures of succinylated proteins can be used to extract more useful information for the characterization of succinylated substrate sites. A stand-alone software will be developed for providing a practical means to facilitate the determination of succinylated targets from a large-scale proteome data.

## Supplementary information

Supplementary figures and tables
