# Supplementary material for: Characterization and Identification of Lysine Succinylation Sites based on Deep Learning Method
Source: Sci Rep. 2019 Nov 7;9:16175. doi: 10.1038/s41598-019-52552-4 (PMC6838336; doi:10.1038/s41598-019-52552-4)
Supplement: Supplementary file 1 — Supplementary figures and tables [file 41598_2019_52552_MOESM1_ESM.docx]

**Supplementary Materials**

**Manuscript Number:** SREP-19-26917

**Manuscript Title:** Characterization and Identification of Lysine Succinylation Sites based on Deep Learning Method
**Authors:** Kai-Yao Huang, Justin Bo-Kai Hsu, and Tzong-Yi Lee

Supplementary Figures

**Figure S1**. **Ten-fold cross-validation performances of CNN models trained using various window lengths (2*n* + 1, *n* is ranging from 5 to 20) based on PspAAC feature.** The CNN models trained with longer sequence lengths (31, 33, and 35 amino acids) can outperform other models. Among these models, the CNN model trained with sequence length of 31 performs best in terms of sensitivity, specificity, and accuracy.

**Figure S2. Comparison of basic architecture between shallow neural network and deep neural network.**

F**igure S3. Comparison of amino acid composition between succinylated sites (blue color) and non-succinylated sites (red color).** This investigation shows that the positively charged lysine (K) residue is remarkably enriched within the neighborhood of succinylated sites.

Figure S4. The comparison of ROC curves among CNN models trained with various attributes.

Figure S5. Performance comparison between our method and existing available succinylation site prediction tools using independent testing dataset.

**Supplementary Tables**

**Table S1. Summary of computational methods dedicated to the prediction of protein succinylation sites.**

**Table S2. Evaluation of ten-fold cross-validation on various machine learning methods trained using individual feature.**


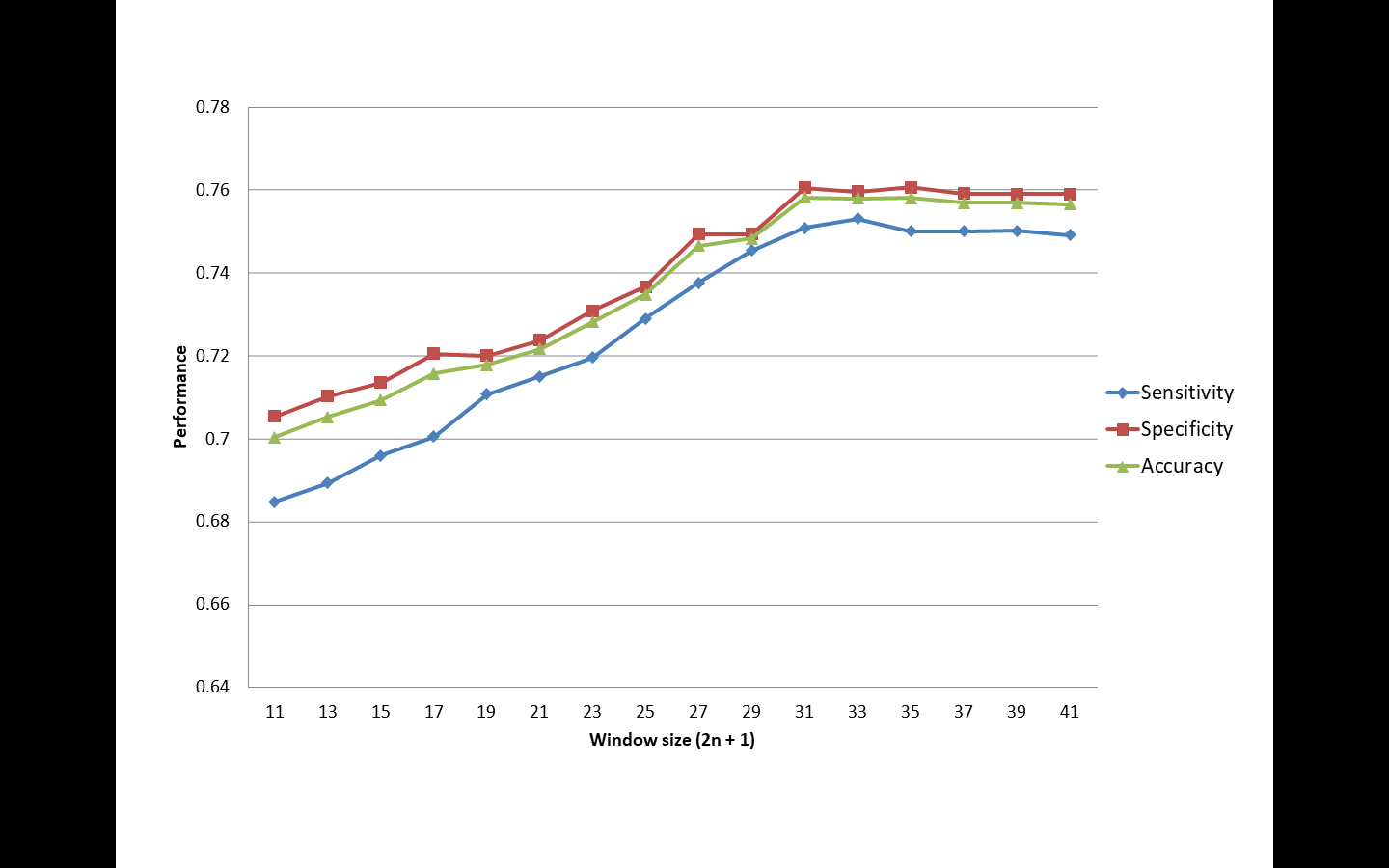


**Figure S1**. Ten-fold cross-validation performances of CNN models trained using various window lengths (2*n* + 1, *n* is ranging from 5 to 20) based on PspAAC feature.


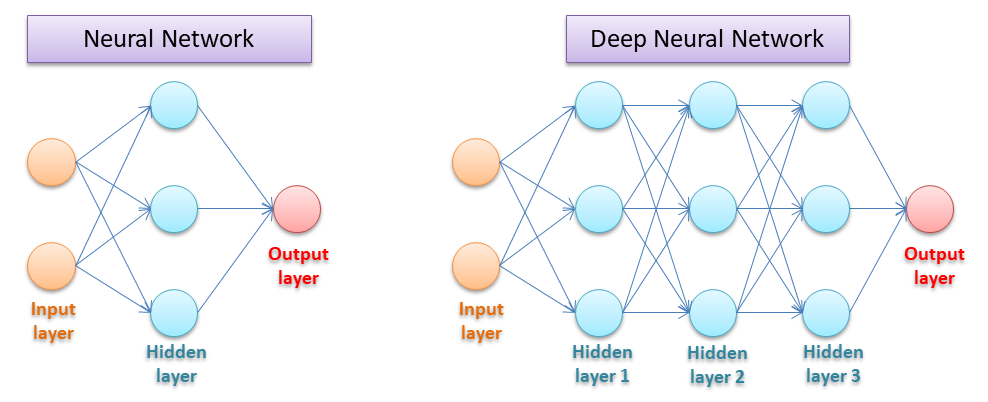


**Figure S2. Comparison of basic architecture between shallow neural network and deep neural network.**


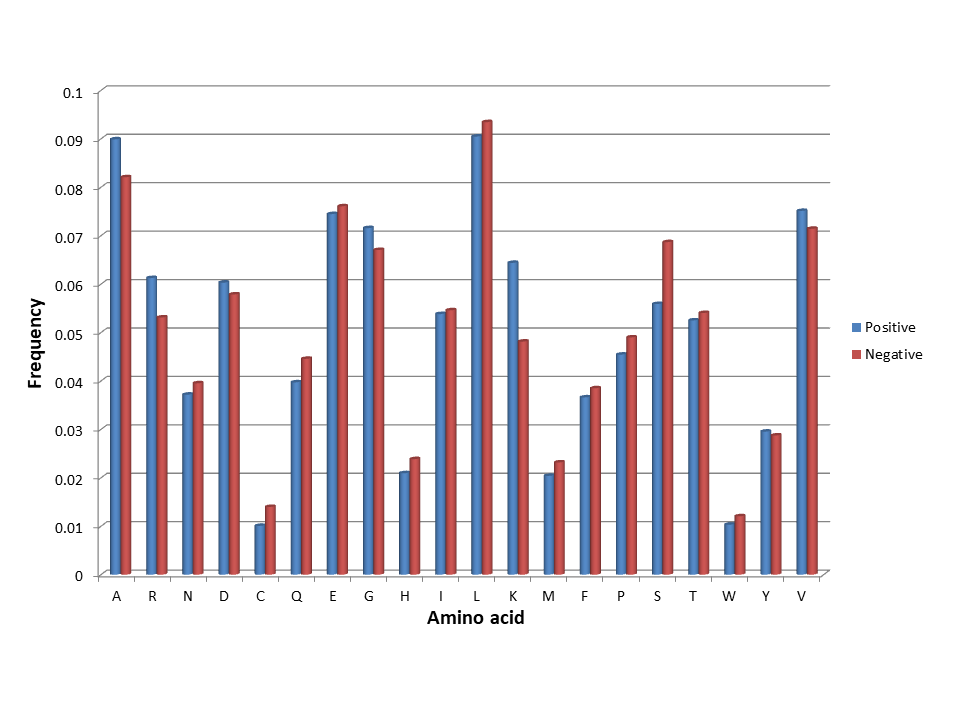


F**igure S3. Comparison of amino acid composition between succinylated sites (blue color) and non-succinylated sites (red color).** This investigation shows that the positively charged lysine (K) residue is remarkably enriched within the neighborhood of succinylated sites.


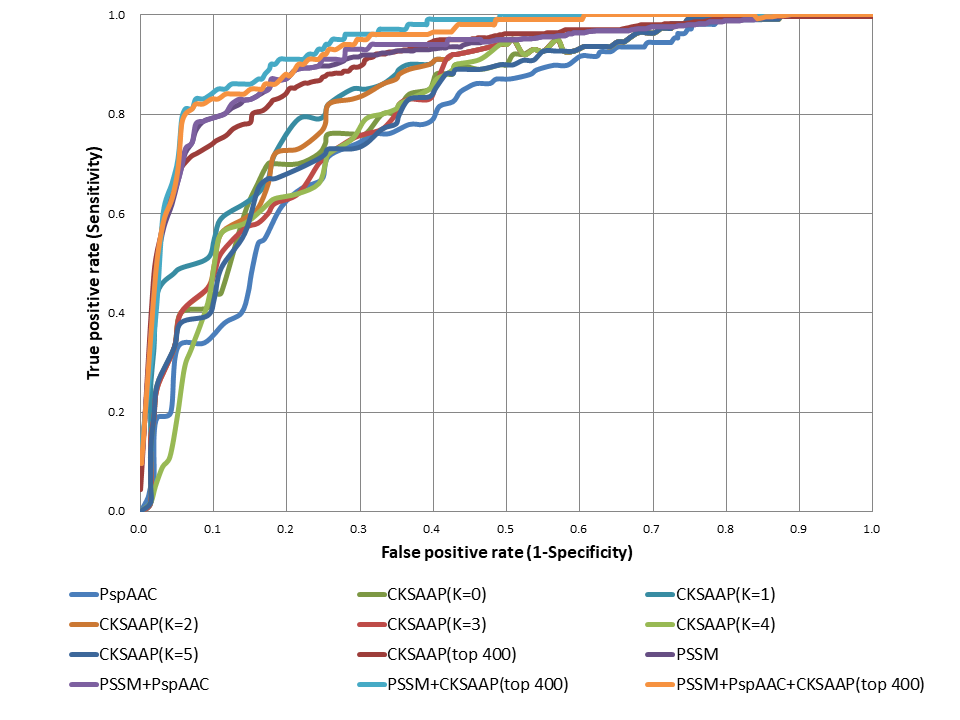


Figure S4. The comparison of ROC curves among CNN models trained with various attributes. In the comparison among single attributes, the values of area under the ROC curve (AUC) are 0.741, 0.772, 0.778, 0.777, 0.770, 0.767, 0.763, 0.839, and 0.858 for PspAAC, CKSAAP(K=0), CKSAAP(K=1), CKSAAP(K=2), CKSAAP(K=3), CKSAAP(K=4), CKSAAP(K=5), CKSAAP(top400), and PSSM, respectively. The AUC values of PSSM+PspAAC, PSSM+CKSAAP(top400), and PSSM+CKSAAP(top400) are 0.867, 0.886, and 0.880, respectively.


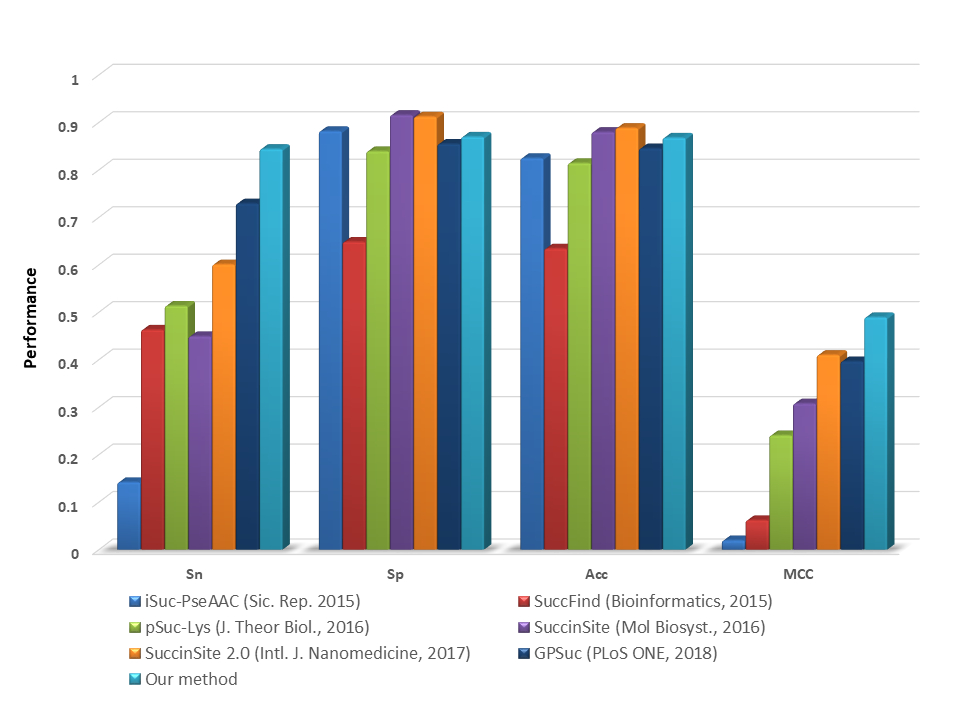


Figure S5. Performance comparison between our method and existing available succinylation site prediction tools using independent testing dataset.

**Table S1. Summary of computational methods dedicated to the prediction of protein succinylation sites.**

| **Method** | **Reference** | **Material** | **Method** | **Attributes** | **Cross-validation (CV)** | **URL link of prediction tool** |
| --- | --- | --- | --- | --- | --- | --- |
| iSuc-PseAAC | *Sci Rep. 2015* | CPLM and UniProtKB | SVM | PseAAC | 10-fold | http://app.aporc.org/iSuc-PseAAC/ |
| SucPred | *J Theor Biol. 2015* | CPLM | SVM | Position weight AAC | 5-fold | Web site is not available |
| iSuc-PseOpt | *Anal Biochem. 2016* | CPLM and UniProtKB | Random forest | PseAAC | 10-fold | http://www.jci-bioinfo.cn/iSuc-PseOpt |
| pSuc-Lys | *J Theor Biol. 2016* | CPLM and UniProtKB | Random forest | PseAAC | 5-fold | http://www.jci-bioinfo.cn/pSuc-Lys |
| SuccinSite | *Mol Biosyst. 2016* | CPLM | SVM | k-spaced amino acid pairs, binary and amino acid index properties | 5-fold | http://systbio.cau.edu.cn/SuccinSite/ |
| SuccFind | *Bioinformatics, 2015* | CPLM and UniProtKB | SVM | Sequence-and evolutionary-derived features | 10-fold | Web site is not available |
| PSSM-Suc | *J Theor Biol. 2017* | CPLM | SVM | PSSM | 6, 8 and 10-fold | https://github.com/YosvanyLopez/PSSM-Suc^d^ |
| SuccinSite2.0 | *International Journal of Nanomedicine, 2017* | CPLM | Random forest | Evolutionary features ,orthogonal binary features | 5-fold | https://biocomputer.bio.cuhk.edu.hk/SuccinSite2.0/ |
| SucStruct | *Analytical Biochemistry, 2017* | CPLM | C4.5 decision tree | Accessible surface area, secondary structure and  local backbone angles | 6, 8 and 10-fold | https://github.com/YosvanyLopez/SucStruct^d^ |
| Success | *BMC Genomics, 2018* | CPLM | SVM | Structural and Evolutionary features | 6, 8 and 10-fold | https://github.com/YosvanyLopez/Success^d^ |
| SSEvol-Suc | *PLOS ONE, 2018* | CPLM | AdaBoost^a^ | Secondary structure feature, evolutionary feature | 6, 8 and 10-fold | https://github.com/YosvanyLopez/SSEvol-Suc^d^ |
| PSuccE | *BMC Bioinformatics , 2018* | UniProtKB and NCBI protein sequence database | SVM | AAC, BE^b^, physicochemical property and GPAAC^c^ | 10-fold | No web site |
| GPSuc | *PLOS ONE, 2018* | SuccinSite2.0 | Random forest | AAC, AAindex, BE, PSSM, and pCKSAAP | 10-fold | http://kurata14.bio.kyutech.ac.jp/GPSuc/ |

^a^AdaBoost: Adaptive Boosting (a meta-classifier)

^b^BE: binary encoding

^c^GPAAC: grey pseudo amino acid composition

^d^Theses methods only provide WEKA command lines instead of the web tool for data submission.

**Table S2. Evaluation of ten-fold cross-validation on various machine learning methods trained using individual feature.**

| **Method** | **Attribute** | **Number of true positives** | **Number of false positives** | **Number of true negatives** | **Number of false negatives** | **Sensitivity** | **Specificity** | **Accuracy** | **MCC** |
| --- | --- | --- | --- | --- | --- | --- | --- | --- | --- |
| Decision tree | PspAAC | 2199 | 5412 | 11000 | 1017 | 68.41% | 67.02% | 67.25% | 0.269 |
| Support vector machine | CKSAAP (top 400) | 2472 | 2900 | 13512 | 744 | 76.87% | 82.33% | 81.43% | 0.491 |
| Random forest | CKSAAP (top 400) | 2535 | 3010 | 13402 | 671 | 78.82% | 81.66% | 81.20% | 0.497 |
| Deep learning | PSSM | 2750 | 2600 | 13812 | 466 | 85.51% | 84.16% | 84.38% | 0.579 |
